# Association of weather and air pollution interactions on daily mortality in 12 Canadian cities

**DOI:** 10.1007/s11869-014-0266-7

**Published:** 2014-05-15

**Authors:** J. K. Vanos, S. Cakmak, L. S. Kalkstein, Abderrahmane Yagouti

**Affiliations:** 1 Environmental Health Research Bureau, Population Studies Division, Health Canada, 50 Columbine Driveway, Ottawa, ON K1A 0K9 Canada; 2 Atmospheric Sciences Group, Department of Geosciences, Texas Tech University, Lubbock, TX USA; 3 Miller School of Medicine, Department of Public Health Sciences, Environment and Public Health Division, University of Miami, Miami, FL USA; 4 Climate Change and Health Office, Health Canada, Ottawa, ON Canada

**Keywords:** Distributed nonlinear lag model, Spatial synoptic classification, Mortality, Air pollution, Heat stress

## Abstract

It has been well established that both meteorological attributes and air pollution concentrations affect human health outcomes. We examined all cause nonaccident mortality relationships for 28 years (1981–2008) in relation to air pollution and synoptic weather type (encompassing air mass) data in 12 Canadian cities. This study first determines the likelihood of summertime extreme air pollution events within weather types using spatial synoptic classification. Second, it examines the modifying effect of weather types on the relative risk of mortality (RR) due to daily concentrations of air pollution (nitrogen dioxide, ozone, sulfur dioxide, and particulate matter <2.5 μm). We assess both single- and two-pollutant interactions to determine dependent and independent pollutant effects using the relatively new time series technique of distributed lag nonlinear modeling (DLNM). Results display dry tropical (DT) and moist tropical plus (MT+) weathers to result in a fourfold and twofold increased likelihood, respectively, of an extreme pollution event (top 5 % of pollution concentrations throughout the 28 years) occurring. We also demonstrate statistically significant effects of single-pollutant exposure on mortality (*p* < 0.05) to be dependent on summer weather type, where stronger results occur in dry moderate (fair weather) and DT or MT+ weather types. The overall average single-effect RR increases due to pollutant exposure within DT and MT+ weather types are 14.9 and 11.9 %, respectively. Adjusted exposures (two-way pollutant effect estimates) generally results in decreased RR estimates, indicating that the pollutants are not independent. Adjusting for ozone significantly lowers 67 % of the single-pollutant RR estimates and reduces model variability, which demonstrates that ozone significantly controls a portion of the mortality signal from the model. Our findings demonstrate the mortality risks of air pollution exposure to differ by weather type, with increased accuracy obtained when accounting for interactive effects through adjustment for dependent pollutants using a DLNM.

## Introduction

Recent studies pertaining to weather and atmospheric effects on humans have established associations between human health outcomes and meteorological conditions (e.g., Basu and Samet 2002; Doyon et al. 2008; Gosling et al. 2009) It is well documented that there is a complex relationship between climate, air pollution, and specific human health outcomes in North America (Hanna et al. 2011; Vanos et al. 2014a; b). Air pollution has been shown in many studies to be a contributing factor to human morbidity and mortality (Hanna et al. 2011; Ramlow and Kuller 1990; Schwartz 1994; Vanos et al. 2014a; b) and is also related to synoptic weather patterns, which cover large regions up to ~1,000 km^2^ (Davis et al. 2010; Greene et al. 1999). Spatial synoptic classification (SSC; Sheridan 2002) allows for the accounting and understanding of how humans respond to a combination of meteorological variables simultaneously. Although temperature alone has been shown to be a satisfactory predictor of health outcomes such as mortality and morbidity (Anderson and Bell 2009; Curriero et al. 2002; Díaz et al. 2005; Hajat et al. 2010), it is known that human physiology responds to the complex synergistic effects of all external elements of ambient air—including air pollution—expressed at the synoptic level as well as by weather type (Greene et al. 1999; Vanos et al. 2014a; Vanos et al. 2014b).

Holistically accounting for all elements of a weather situation is important to provide more accurate results in modeling. Time series models have become more prevalent in literature as a means to measure the short-term health effects of weather as well as modifying and synergistic effects of such atmospheric variables (e.g., Anderson and Bell 2009; Bell et al. 2004; Cakmak et al. 2010; Dominici et al. 2006; Guo et al. 2011; Samet et al. 2000a; b). For example, using a case-crossover approach (Stafoggia et al. 2008) estimated the effect of particulate matter <10 μm (PM_10_) on mortality to be greater on days with higher temperature, indicating positive interactions, yet were commonly not statistically significant. Anderson and Bell (2009) made use of a Poisson generalized additive model demonstrating an increase of 3.0 % in the risk of death from high temperatures in 107 US communities. Yet when controlling for the confounding factor of air pollution (ozone (O_3_) and particulate matter <10 μm (PM_10_)), they found the model signal to be slightly lowered (~0.4 % on average) due to the interactions between weather and air pollution on mortality; however, this was not significant, and little air pollution data were available in the chosen communities. Additionally, Bell et al. (2004) found that when using both constrained and unconstrained distributed lag models (DLMs), a 10-ppb increase in O_3_ was associated with an 0.52-% increase in daily mortality based on 7-day delayed effects.

Conventional DLMs rely on the assumption of a linear effect between exposure and outcome; however, accounting for the known nonlinear relationships is vital (Braga et al. 2001; Gasparrini and Armstrong 2010; Gasparrini 2011). Further, using only exposure-response curve methods does not capture the nonlinear associations and can create difficulties when comparing disparate climates across the country (Anderson and Bell 2009). The delayed effects of temperature and/or air pollution across a range of values and time lags can be modeled using a distributed lag nonlinear model (DLNM) (Armstrong 2006; Gasparrini and Armstrong 2010; Gasparrini 2011) with sufficient sample sizes present. Additional effects rather than just temperature are also present, which are not captured in merely temperature-mortality models, and further studies are needed to evaluate these additional effects (e.g., duration, lags, air pollution) (Rocklov et al. 2012).

There remains a considerable amount to discover concerning the modifying effects of weather types and specific variables on air pollution-health interactions (Dear et al. 2005; Hajat et al. 2010). As the urban mix of air pollutants is complex, more careful studies needed to determine which aspects are most harmful to humans (Brook et al. 2007). Given the variety of weather elements affecting pollution levels, a synoptic meteorology approach is well-suited to study city-specific pollution variations (Greene et al. 1999; Rainham et al. 2005). Rainham et al. (2005) found minimal consistency in the pattern between health effects of air pollution during both the summer and winter seasons in Toronto, Canada, yet pollution concentrations were dependent on weather type.

Inconsistencies in results also necessitate further research into air pollution and interactive effects relating climate to mortality. Despite the long-standing research on potential human health impacts due to changing temperatures, along with future climate change predictions (Dessai 2003; Gosling et al. 2009, 2012; Greene et al. 2011), there has been minimal application of synoptic weather types to climate-air pollution interactions with mortality. Accordingly, the goal of this study is to conduct a large-scale investigative analysis, using 28 years of data (1981–2008) for the summer season (Jun-Jul-Aug; JJA), assessing the short-term effects of exposure to the air pollutants nitrogen dioxide, ozone, sulfur dioxide, and particulate matter <2.5 μm on the relative risk of mortality (RR). We examine the modifying effects of weather type on RR using a daily spatial synoptic classification (SSC; Sheridan and Dolney 2003; Sheridan 2002). Modifying effects are first investigated with a single-pollutant model. We then adjust these effects for the remaining three pollutants to determine dependent pollutant effects on mortality risk. Both city-specific and pooled relative risks of mortality are calculated. Urban variations of extreme pollution levels in the 12 large Canadian cities are also assessed based on SSC weather type.

## Methods

### Mortality data

Daily nonaccidental-related mortality data from 1981–2008 were obtained from the Canadian Vital Statistics databases at Statistics Canada (via the Public Health Agency of Canada) for Vancouver, BC; Calgary, AB; Edmonton, AB; Regina, SK; Winnipeg, MB; Toronto, ON; Windsor, ON; Ottawa, ON; Montreal, QC; Quebec City, QC; Halifax, NS; and St. John’s, NF. Data availability for Quebec City and Montreal extend only to the year 2000; hence, we complete the analysis for these cities for 20 rather than 28 years. Causes of death were categorized using codes from the International Classification of Diseases (ICD) 9th revision (codes <800) and ICD 10th revision (codes A00 to R99) (World Health Organization WHO 2010). All nonaccidental mortality is considered, rather than only heat-related, as significant undercounts by the coroner when identifying heat deaths commonly occur (Dixon et al. 2005). The mortality rate per 100,000 people was calculated based on yearly population values for each city accessed from Statistics Canada.

### Spatial synoptic classification data

A suite of routinely monitored meteorological parameters is used to identify each weather situation as one of six weather-type categories, plus a transition category (where one weather type yields to another). The current study focuses on the five warm or hot weather types, with descriptions specific to Canadian summers as follows (see Rainham et al. (2005) for further detail):

#### Dry moderate (DM)

A pleasantly warm, dry weather type that is common throughout much of southern Canada in the summer and is associated with mostly sunny conditions.

#### Dry tropical (DT)

The combined hottest and driest weather type, with sunny, clear skies and occurs subsequent to, or during, anticyclone events with large regional subsidence of air.

#### Moist moderate (MM)

Mild, humid, cloudy, unstable weather often bringing rain showers and occurs with warm fronts or from modification of moist tropical air in the summer.

#### Moist tropical (MT)

Combined hottest and most humid weather type. Skies are partly cloudy in the summer due to instability and convection.

#### Moist tropical plus (MT+)

Extreme subset of MT, in which morning and afternoon apparent temperatures are both above the corresponding MT mean temperatures for the specific location.

Developed by Sheridan (2002), the SSC is a semi-automated classification system that derives from an algorithm comparing listed surface observations to days that are most representative of the various weather types at each monitoring station. The SSC is based on “sliding seed days” representing expected and observed meteorological conditions at each location throughout the year for each weather type. To select seed days for each season and location, thresholds for typical weather variables are quantified for each weather type (i.e., air and dew point temperatures, air pressure, wind velocity, cloud cover), which define that weather type for the given location and time of year. Each day can then be classified into one of the six weather types based on representativeness of the local climate (see Sheridan (2002) and Hondula et al. (2013) for specific methodology). The SSC accounts for relative temporal and spatial variability and hence lends itself well to understanding city-specific health outcomes. For example, a summer MT+ day in Toronto is warmer (0300 h *T*_avg_ = 30.3 °C) than a summer DT day in Quebec City (*T*_avg_ = 28.6 °C) (see Table 1).

**Table 1:** Descriptive summertime statistics for 12 Canadian cities within five weather types (1981–2008): weather type frequency for Jun-Jul-Aug (JJA) (%), relative mortality, 2011 population, mean 1600 h air temperature (T_a_), and ambient pollution concentrations (NO_2_, O_3_, SO_2,_ PM_2.5_)

City population	Synoptic category	Frequency (%)	Relative mortality	SD	Air temp (T_a_) (°C)	SD	NO_2_ (ppb)	SD	O_3_ (ppb)	SD	SO_2_ (ppb)	SD	PM_2.5_ (μg m^−3^)	SD
Calgary 1,214,839	DM	33.4	1.10	0.38	24.07	3.03	19.66	6.15	25.03	6.30	2.34	1.92	9.95	9.16
	DT	3.2	1.11	0.38	29.61	2.22	21.77	5.29	26.79	6.20	2.84	2.05	17.11	16.69
	MM	11.9	1.11	0.35	18.72	2.88	18.07	6.37	22.99	5.91	2.09	1.71	8.27	4.20
	MT	0.5	1.27	0.35	27.41	2.78	17.14	5.66	26.61	4.88	2.50	2.02	12.88	3.64
	MT+	0.0	NA		NA		NA		NA		NA		NA	
Edmonton 1,159,869	DM	19.5	1.37	0.48	24.67	2.84	16.13	8.62	28.60	7.39	1.86	1.49	10.73	9.54
	DT	1.1	1.36	0.57	28.88	3.07	15.64	10.54	35.50	8.71	1.44	1.28	12.50	5.16
	MM	8.1	1.38	0.49	19.78	2.72	12.57	6.08	23.81	6.26	1.43	1.14	7.81	6.37
	MT	0.7	1.42	0.38	27.78	1.87	10.10	5.82	33.49	4.71	1.30	0.67	11.91	3.04
	MT+	0.0	NA		NA		NA		NA		NA		NA	
Halifax 390,328	DM	21.1	3.03	0.95	22.95	2.40	14.63	8.22	20.79	8.38	6.83	4.19	7.39	3.92
	DT	0.6	3.19	0.81	29.50	2.22	16.78	6.03	24.19	8.17	7.95	4.25	10.17	5.49
	MM	23.7	2.91	0.94	19.84	2.52	14.42	9.62	20.72	9.97	6.53	4.35	7.69	3.98
	MT	7.7	2.99	0.97	25.21	2.23	15.04	10.53	23.25	10.12	6.51	4.37	11.98	7.96
	MT+	1.2	2.77	0.89	27.21	2.14	14.46	10.40	18.87	8.94	6.42	6.28	16.50	10.82
Montreal 3,824,221	DM	32.2	1.96	0.36	25.46	2.23	21.39	7.19	19.68	7.79	4.27	2.64	8.32	4.42
	DT	1.3	2.27	0.53	30.59	1.62	24.52	7.71	47.17	17.49	6.75	5.18	11.20	3.44
	MM	24.0	1.95	0.36	21.70	2.33	19.73	6.49	17.25	7.44	3.18	2.22	9.54	5.23
	MT	22.0	2.13	0.42	27.80	2.37	22.89	8.19	28.30	10.59	3.65	2.40	16.52	9.18
	MT+	4.4	2.38	0.59	30.35	1.90	24.61	10.84	34.12	11.11	3.68	2.38	20.20	11.99
Ottawa 1,236,324	DM	29.7	1.41	0.44	25.55	2.37	15.23	8.49	20.31	8.11	2.52	2.32	6.99	6.15
	DT	3.7	1.45	0.50	31.61	1.86	19.69	10.71	33.37	12.35	3.39	2.53	13.72	6.06
	MM	24.3	1.40	0.44	22.11	2.68	15.59	8.74	20.37	8.18	2.01	2.07	8.53	5.98
	MT	16.2	1.43	0.46	28.37	2.32	18.54	10.13	28.53	11.02	2.64	2.28	15.47	8.65
	MT+	2.4	1.56	0.48	30.86	2.14	17.12	11.03	35.13	12.05	2.99	2.73	18.96	10.13
Quebec City 3,824,221	DM	30.8	1.64	0.58	24.33	2.69	14.38	8.44	18.33	7.09	3.44	7.42	8.52	5.04
	DT	0.6	1.69	0.65	29.30	2.49	19.09	11.33	22.58	15.33	7.10	8.95	NA	NA
	MM	22.8	1.63	0.57	20.31	2.65	13.70	9.11	16.63	7.41	5.55	13.57	7.20	3.65
	MT	16.1	1.76	0.67	26.68	2.59	14.78	8.89	24.53	11.16	2.70	5.45	19.50	8.98
	MT+	2.9	2.00	0.81	29.21	2.38	13.17	7.55	28.58	14.51	1.75	2.40	21.75	11.73
Regina 210,556	DM	30.2	1.40	0.81	26.11	2.94	14.27	11.48	25.34	6.57	0.68	0.86	8.74	4.38
	DT	4.0	1.34	0.79	32.57	2.60	19.10	15.48	31.80	7.93	1.07	1.29	10.29	5.34
	MM	11.2	1.38	0.80	21.68	2.59	11.49	8.13	23.31	6.37	0.42	0.70	6.91	3.53
	MT	1.1	1.41	0.81	28.19	3.08	12.51	9.56	27.93	7.51	0.50	0.66	10.31	3.60
	MT+	1.4	1.43	0.61	31.84	2.41	11.64	8.14	30.91	8.71	0.56	0.72	13.60	3.64
St. John 210,556	DM	19.4	1.84	0.87	20.43	2.64	6.15	4.01	21.08	8.70	3.31	2.84	5.23	2.68
	DT	0.0	NA		NA		NA		NA		NA		NA	
	MM	23.0	1.80	0.87	17.78	2.62	6.84	5.42	21.11	10.17	3.20	2.93	5.15	3.84
	MT	6.9	1.85	0.89	22.77	1.78	4.79	4.78	20.88	10.25	2.49	2.54	5.87	4.62
	MT+	1.3	2.04	0.89	23.83	1.53	3.15	2.79	17.08	10.93	1.69	1.84	6.09	8.01
Toronto 5,583,064	DM	34.2	1.56	0.27	24.93	2.40	22.77	8.10	24.68	7.67	3.17	2.60	8.48	5.50
	DT	6.2	1.62	0.31	31.67	2.15	27.29	9.11	42.08	11.07	4.84	3.11	20.69	11.03
	MM	18.8	1.58	0.29	21.35	2.52	23.30	7.99	23.05	8.46	2.69	2.09	11.87	7.33
	MT	21.5	1.62	0.28	27.50	2.55	24.49	7.96	33.09	8.90	3.83	2.73	19.00	9.17
	MT+	3.5	1.68	0.31	30.18	2.36	23.45	7.47	28.48	8.48	3.94	3.13	25.19	9.36
Vancouver 2,313,328	DM	48.9	1.54	0.33	21.27	2.23	17.77	6.68	17.67	4.72	4.13	2.34	6.31	2.83
	DT	0.0	NA		NA		NA		NA		NA		NA	
	MM	24.3	1.50	0.31	19.00	1.64	15.43	4.81	13.56	4.30	3.55	1.91	5.53	2.64
	MT	0.7	1.77	0.26	26.91	1.56	24.53	7.60	29.75	5.09	5.70	2.37	10.45	2.09
	MT+	0.0	NA	NA	NA		NA		NA		NA		NA	
Windsor 319,246	DM	28.0	1.43	0.76	26.77	2.23	22.12	9.40	32.83	10.46	6.60	4.48	11.33	6.74
	DT	4.7	1.46	0.72	32.54	2.06	23.81	11.09	46.93	12.07	9.95	5.12	22.16	11.11
	MM	19.4	1.45	0.73	22.96	2.33	20.88	9.51	26.39	8.86	5.13	3.89	12.24	6.60
	MT	29.1	1.45	0.77	28.69	2.63	20.90	9.50	37.43	10.47	6.47	3.92	19.72	9.03
	MT+	7.3	1.55	0.83	31.27	2.22	20.47	9.00	39.92	9.98	6.55	3.90	23.74	11.01
Winnipeg 730,018	DM	28.9	1.91	0.57	26.01	2.76	11.48	5.24	22.20	6.72	0.73	1.40	7.42	5.18
	DT	1.9	1.99	0.59	32.64	2.89	12.69	5.24	34.26	9.71	0.95	1.02	10.60	2.43
	MM	17.4	1.96	0.60	21.99	2.66	9.86	4.17	19.58	6.74	0.48	0.94	5.88	3.17
	MT	15.1	1.97	0.62	28.08	2.66	10.12	4.12	27.33	7.20	0.41	0.70	8.49	3.79
	MT+	2.9	1.96	0.59	30.79	2.32	9.18	3.59	30.92	7.96	0.57	1.26	9.26	4.15
Average	DM	29.8	1.68	0.49	24.38	1.95	16.33	4.80	23.04	4.45	3.32	1.96	8.28	1.78
	DT	2.5	1.75	0.60	30.89	1.49	20.04	4.42	34.47	8.75	4.63	3.18	14.27	6.27
	MM	19.1	1.67	0.46	20.60	1.60	15.16	4.76	20.73	3.58	3.02	1.94	8.05	2.26
	MT	11.5	1.75	0.47	27.12	1.65	16.32	6.27	28.49	4.69	3.22	2.10	13.51	4.54
	MT+	2.3	1.92	0.41	29.51	2.38	16.32	7.38	30.99	7.70	3.47	2.39	17.25	8.21

Mortality rate per 100,000 people, calculated based on yearly population; Statistics Canada 2011 Census, population for census metropolitan area

Meteorological data used to classify weather types into SSC categories for each of the 12 cities are obtained from first-order airport weather stations maintained by the Meteorological Service of Canada. Table 1 lists the city-specific information pertaining to each weather type, including the summertime frequencies, mean 1500 h air temperatures, and air pollution concentrations. The SSC has been implemented in many human health-climate studies (e.g., Hajat et al. 2010; Hanna et al. 2011; Vanos et al. 2013a; b; Sheridan et al. 2009). The weather types most commonly associated with increases in mortality (and therefore designated as “offensive” weather types) are MT+ and DT (Sheridan and Kalkstein 2004).

### Air pollution data

Air pollution data from the National Air Pollution Surveillance Network (NAPS) database were collected for the period of 1981–2008. Measurements of average hourly concentrations of ozone (O_3_, ppb), nitrogen dioxide (NO_2_, ppb), particulate matter <2.5 μm in diameter (PM_2.5_, μm m^−3^), and daily sulfur dioxide (SO_2_, ppb) were utilized in this analysis. Hourly measurements were averaged to obtain daily concentrations of these pollutants. For PM_2.5_, the earliest year for which data were available was 1998. For both SO_2_ and O_3_, complete datasets were available for the 1981–2008 period for all cities except St. John’s (data from 1989–2008). NO_2_ data were complete for all cities in the given years except for Calgary and St. John’s (available data beginning in 1990 and 1989, respectively). Days with no recorded data were considered as missing data and treated as such in the model.

### Data analysis

#### Extreme pollution episodes

A detailed evaluation of the synoptic conditions associated with extreme air pollution levels for each city is completed using a method by Greene et al. (1999), whereby the top 5 % of pollution days (classified as “extreme”) for each pollutant is examined by air mass (or weather type). To do so, we determine the relative ratio representing the relationship between the percent of days in the top 5 % of pollution levels, to the percent frequency of the select weather type. Therefore, a ratio of 1.0 indicates that an extreme pollution episode is more likely to occur in the given weather type. A ratio ≥2.0 indicates statistical significance (*p* < 0.05) of this likelihood (Greene et al. 1999; Kalkstein 1991). A value of “0.0” for a particular pollutant signifies virtually no chance of the level of the given pollutant reaching the highest 5 % level based on the data records.

#### Distributed lag nonlinear modeling

For each city and weather type, the RR due to exposure to each air pollutant is modeled using a DLNM. In addition, the interactive effects of exposure to two pollutants are modeled to determine the modifying effect on risk of mortality. The DLNM is a relatively new standardized approach that uses a time series modeling framework to describe the simultaneous nonlinear and delayed effects between predictors and an outcome (Gasparrini 2011). The overarching purpose is to provide a statistical regression model that defines the relationship between a set of predictors and an outcome and then to estimate the risk effect (Gasparrini 2011). The DLNM also has the advantage of using more than one variable showing delayed effects (e.g., two-pollutant interactive effects) to be transformed through a cross-basis function. The framework can describe flexible relationships based on air pollution as a predictor, and the dimension of the lag, hence, specifying two independent predictors (Gasparrini and Armstrong 2010). We apply lags of 0–6 days for each pollutant examined to estimate the RR due to exposure in a single day (lag 0) and multiple lag days, where the total estimate by the DLNM summarizes the effects of cumulative exposure over the previous days (Dominici et al. 2006). This addresses the further complexity that arises when there are delayed effects of a predictor (air pollution) on an outcome (mortality). Specific details and examples are provided in Gasparrini and Armstrong (2010, 2012) and Gasparrini (2011).

In order to remove any temporal variability (cycles) in mortality, we adjust for various time confounders using a categorical variable for day of week (DOW) and apply a natural cubic spline of time with one knot at each of 30, 120, 180, and 365 days of observation for monthly, 3-month (seasonal), 6-month (biannual), and yearly time effects, respectively. Separate model runs are completed for exposure to single air pollutants as well as adjustments for the single pollutant due to simultaneous exposure to the remaining three pollutants; hence, all two-way interactions were assessed. The optimal number of knots is determined based on the Akaike information criterion (AIC) goodness-of-fit test, the Bartlett test for autocorrelation, as well as visual examination to maximize the evidence that the residuals do not display any type of structure. Within the DLNM, we control for mean air temperature, rather than minimum or maximum, as preliminary analysis demonstrated enhanced model strength based on model prediction values (or AIC). This was also found to be true based on AIC testing by Curriero et al. (2002) in 11 US cities, with further studies (Bell et al. 2004; Guo et al. 2011; Samet et al. 2000a; b) also using mean temperature based on extensive work and thereby accounting more fully temperatures experienced throughout the full day, rather than at one time. As an example, the pollutant-adjusted models can be summarized generally as follows:

1 $$ LogE\left({Y}_t\kern0.5em /{X}_t\right)\sim \kern0.5em \beta {X}_{t-1}+\delta {X^{,}}_{t-1}+{\mathrm{DOW}}_t\kern0.5em +\mathrm{ns}\left({T}_{\mathrm{avg},} df\right)+\mathrm{ns}\left(\mathrm{time}, df\right) $$

where *Y*_*t*_ is the daily count of nonaccidental mortality, *X*_*t*−*l*_ is the main pollution level on day *t* with 0 to 6 days of lag, *X*’ is the adjusting pollutant level at same lag, and *β* and *δ* are the regression coefficients linking the main pollutant and the adjusting pollutant to daily mortality, respectively, DOW_*t*_ indicates the day of the week on day *t*, ns(*T*_avg_, 4) is the natural spline of temperature with four degrees of freedom (*df*), ns(time, *df*) is the natural spline of calendar time with *df* corresponding to a knot 30, 120, 180, and 365 days of observation. The effect estimates for each season and weather type were obtained by pollutant × season / weather type interaction terms (Vanos et al. 2014a; Vanos et al. 2014b). For example, season-specific DM effect estimates can be found by replacing *β* by *β*_(w,dm)_I_w_I_dm_ + *β*_(sp,dm)_I_sp_I_dm_ + *β*_(au,dm)_I_au_I_dm_ + *β*_(su,dm)_I_su_I_dm_, where I_w_, I_sp_, I_au_, and I_su_ are indicators of winter, spring, autumn, and summer, respectively, and I_dm_ is indicator of DM weather type. The selected models for each city are pooled into one estimate using a random effects model. From this, we present the increase in relative risk of mortality (RR) for an interquartile increase of exposure to each individual pollutant and each two-way interaction, with 95 % confidence intervals (CI).

Based on standard deviations and the upper and lower 95 % CI, we are able to describe any uncertainty and variability in the estimates of RR. Single and pooled RR estimates are tested for statistical significance using *t* tests, and a level of statistical significance of 0.05 (where a *t* value >2.0 indicates a statistically significant positive relationship). Pearson correlation coefficients (*r*) are calculated to determine the long-term correlations among each pair of air pollutants, as well as air and dew point temperatures, within each weather type. Statistical modeling and analyses were completed in R version 2.14.1 (The R Foundation for Statistical Computing, 2012), using the DLNM package by Gasparrini and Armstrong (2012).

## Results

Across Canada, the prevalence of the mild and benign DM weather type in the summer season is the most prevalent (Table 1), with DT being the least common weather type (highest prevalence in Toronto (6.7 %); very rare in the coastal cities of Halifax, St. John’s, and Vancouver). The DT weather type has the highest mean air temperature coinciding with the highest air pollution concentrations of NO_2_, O_3_, and SO_2_. Moist tropical plus (MT+) presents the second highest air temperatures and the highest concentrations of PM_2.5_. MT overnight temperatures (0300 h) are generally the highest of all the summer air masses.

The extreme air pollution and temperature conditions in the MT and MT+ weather types align closely with a consistently elevated relative mortality, with MT+ displaying the highest overall mean standardized mortality for all cities (1.92). The DT and MT weather types have an average mortality rate of 1.75 deaths per 100,000 people. The most benign weather types are shown to be DM and MM, demonstrating the lowest standardized mortality and air temperatures, as well as the lowest and most comparable concentrations of all pollutants. However, air pollution levels and rates of mortality vary by city. For example, O_3_ and NO_2_ concentrations in the DT weather type reach 46.93 and 23.81 ppb, respectively, in Windsor, 47.17 and 24.52 ppb in Montreal, yet only 31.80 and 19.1 ppb in Regina. In the listed cities, the respective rates of mortality (±SD) are 1.46 ± 0.72, 2.27 ± 0.53, and 1.34 ± 0.79.

The long-term air pollution correlations within each examined weather type are displayed in Table 2. Nitrogen dioxide is significantly correlated with SO_2_ and PM_2.5_ in all weather types, with the strongest relationships found in the hottest weather types. Ozone is found to correlate only with PM_2.5_, where moderate and significant correlation coefficients exist between the two in all weather types. One exception is a moderate O_3_-SO_2_ correlation in DT weather, which can be explained by increased sunlight, heat, and stability in the hot and dry air (He and Lu 2012). Sulfur dioxide is significantly correlated to both NO_2_ and PM_2.5_, with the strongest relationships again found within the two most oppressive weather types (DT, MT+) and the weakest in MM air. Significant correlations of PM_2.5_ with the remaining pollutants are generally present in all weather types, being the weakest in DM and MM. Overall, the strongest relationship among air pollutants is found in the hot, dry DT weather type. Further, air temperature has significant and moderate correlations with O_3_ and PM_2.5_ in many of the weather types and is strongest in the hot weather types of DT and MT+.

**Table 2:** Matrix of Pearson correlation coefficients between air pollutants, air temperature (*T*_a_), and dew point temperature (*T*_d_) in select weather types during the summer season, 1981–2008 for 12 Canadian cities

DM	NO_2_	O_3_	SO_2_	PM_2.5_	*T*_a_	*T*_d_
NO_2_	1					
O_3_	0.10	1				
SO_2_	0.36^†^	0.05	1			
PM_2.5_	0.37^†^	0.38^†^	0.25^†^	1		
*T*_a_	0.20^†^	0.29^†^	0.11	0.33^†^	1	
*T*_d_	0.07	0.02	0.16	0.18	0.47^†^	1
DT	NO_2_	O_3_	SO_2_	PM_2.5_	*T*_a_	*T*_d_
NO_2_	1					
O_3_	0.13	1				
SO_2_	0.50^†^	0.32^†^	1			
PM_2.5_	0.43^†^	0.50^†^	0.36^†^	1		
*T*_a_	0.08	0.51^†^	0.21^†^	0.23^†^	1	
*T*_d_	0.06	0.43^†^	0.25^†^	0.28^†^	0.66^†^	1
MM	NO_2_	O_3_	SO_2_	PM_2.5_	*T*_a_	*T*_d_
NO_2_	1					
O_3_	0.05	1				
SO_2_	0.19^†^	0.04	1			
PM_2.5_	0.44^†^	0.44^†^	0.20^†^	1		
*T*_a_	0.18	0.20^†^	0.00	0.40^†^	1	
*T*_d_	0.13	0.10	0.07	0.38^†^	0.58^†^	1
MT	NO_2_	O_3_	SO_2_	PM_2.5_	*T*_a_	*T*_d_
NO_2_	1					
O_3_	0.06	1				
SO_2_	0.39^†^	0.17	1			
PM_2.5_	0.50^†^	0.60^†^	0.37^†^	1		
*T*_a_	0.23^†^	0.32^†^	0.12	0.42^†^	1	
*T*_d_	0.17	0.08	0.12	0.38^†^	0.32^†^	1
MT+	NO_2_	O_3_	SO_2_	PM_2.5_	*T*_a_	*T*_d_
NO_2_	1					
O_3_	0.11	1				
SO_2_	0.41^†^	0.02	1			
PM_2.5_	0.54^†^	0.34^†^	0.39^†^	1		
*T*_a_	0.05	0.49^†^	0.09	0.26^†^	1	
*T*_d_	0.06	0.34^†^	0.07	0.26^†^	0.79^†^	1

^†^ *p* < 0.05 level of significance

### Extreme air pollution likelihoods

We have identified the synoptic conditions (weather types) that when present, have a statistically significantly higher likelihood of being associated with extreme pollution episodes that have been shown to harm human health (Greene et al. 1999) (Table 3). Results for all cities combined demonstrate that on average, extreme air pollution episodes (defined as significantly elevated levels of NO_2_, O_3_, SO_2,_ and PM_2.5_) are most likely to occur in the DT weather type, with relative likelihood ratios of 2.1, 6.0, 2.2, and 4.2, respectively. Significant combined city results are also prevalent in hot, humid weather. For MT weather, extreme pollution episodes due to O_3_ or PM_2.5_ are almost three times more likely than normal. For MT+, the resulting ratios are 3.8 for O_3_ and 2.8 for PM_2.5_. Overall, 75 and 67 % of the cities studied experience a statistically significant likelihood of extreme episodes of O_3_ and PM_2.5_ pollution, respectively, under the DT weather type. Those cities that do not include coastal maritime cities (Vancouver, Halifax, and St. Johns) experience little to no dry, hot (DT) weather, and hence, no analysis can be completed under this weather type in such climates; rather, in these coastal cities, it is the more humid MT weather type that significantly raises the likelihood of O_3_ and PM_2.5_ extreme pollution events. It is worth mentioning here that the DT weather type is present an average of 2.3 % (0–6.2 %) of summer days, and the MT and MT+ weather types only 11.5 % (0.5–29.5 %) and 2.3 % (0–7.3 %), respectively, of summer days in the studied cities.

**Table 3:** Summertime (JJA) synoptic weather types per city reported based on frequency (%) and likelihood ratio to result in extreme pollution episodes. Select weather types chosen based on high ratios and weather type presence

City	Category	Freq	Pollutant Ratio^a^			
		(%)	NO_2_	O_3_	SO_2_	PM_2.5_
Calgary^b^	DT	3.2	1.93	*2.17*	*2.21*	*5.56*
	MT	0.5	1.54	1.54	*3.13*	*3.95*
	DM	33.4	1.35	1.63	1.09	1.79
Edmonton^b^	DT	1.1	1.43	*8.57*	1.47	*5.48*
	MT	0.7	0.00	*2.35*	0.00	*6.01*
	DM	19.5	1.67	*2.86*	0.86	*2.03*
Halifax	DT	0.6	1.82	1.66	1.74	5.73
	MT	7.7	1.17	1.65	0.37	*2.04*
	MT+	1.2	1.29	0.74	1.54	*2.55*
Montreal	DT	1.3	1.82	*10.91*	*2.43*	*8.49*
	MT	22	0.89	1.7	0.89	1.88
	MT+	4.4	0.89	2.83	0.53	*3.97*
Ottawa	DT	3.7	*2.37*	*5.47*	*2.32*	1.13
	MT	16.2	1.54	1.65	0.97	*3.00*
	MT+	2.4	0.68	1.67	0.33	1.79
Quebec City	DT	0.7	*2.88*	*5.23*	*2.82*	NA
	MT	16.1	1.07	*2.30*	0.58	*2.95*
	MT+	2.9	0.30	*3.04*	0.00	*3.19*
Regina	DT	4.0	*3.04*	*4.84*	1.11	*2.07*
	MT	10.9	1.01	*2.21*	0.08	0.75
	MT+	1.4	1.47	*4.27*	0.00	*2.00*
St. John^c^	MT	6.9	0.5	*2.01*	0.56	*2.86*
	MT+	1.3	0.00	0.98	0.00	*3.09*
	MM	23.0	0.53	1.92	1.01	1.55
Toronto	DT	6.2	1.63	*7.52*	*2.76*	*5.43*
	MT	21.5	1.20	1.41	1.12	1.66
	MT+	3.5	0.66	1.32	0.66	*3.93*
Vancouver^b,c^	MT	0.7	*3.15*	*13.64*	*2.1*	*5.88*
	DM	21.5	1.60	1.48	1.29	1.29
	MM	3.5	1.40	0.75	1.83	*2.11*
Windsor	DT	4.7	*2.40*	*6.18*	*3.12*	*3.73*
	MT	26.3	0.71	0.99	0.59	1.26
	MT+	7.3	0.22	0.65	0.11	1.82
Winnipeg	DT	1.9	1.63	*7.86*	1.88	0.00
	MT	15.1	0.58	1.62	0.00	1.81
	MT+	2.9	0.00	*2.35*	0.00	*2.50*

^a^Ratio = (% of days within the top 5 % level of pollution):(overall/% of occurrence of the weather type in JJA). A ratio >2.0 (italics) identifies those synoptic categories where the occurrence of an extreme pollution episode is statistically significantly more likely to occur, being greater than expected (Greene et al. 1999)

^b^MT+ weather type not present

^c^DT weather type not present

Vancouver is quite exceptional among the 12 cities, with a significantly greater frequency of extreme pollution episodes for all pollutants studied (ratio = 4.17 on average) when the MT weather type is present. The MT+ weather type yields similar results wherever it is present, but occurs very infrequently in many areas of Canada. In Toronto, Winnipeg, Ottawa, Montreal, and Quebec City, the greatest extreme episode frequencies are associated with DT (ratio = 4.20 on average). A similar result occurs farther west (e.g., Regina, Edmonton, Calgary), where the average likelihood ratio is 3.20. Moderate weather types (DM and MM) show very minimal chance of extreme pollution episodes.

### DLNM results

**Fig. 1:**
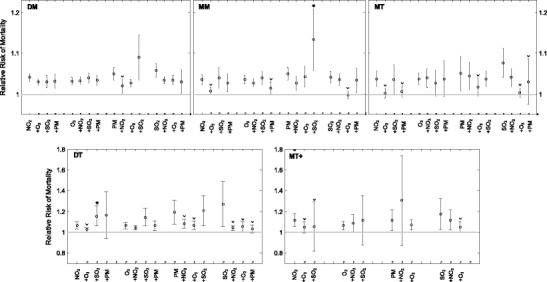
Relative risks of mortality with 95 % confidence intervals (CI) within five weather types and *n* number of cities valid per weather type: dry moderate (*DM*) (*n* = 12), moist moderate (*MM*) (*n* = 12), dry tropical (*DT*) (*n* = 5), moist tropical (*MT*) (*n* = 8), and moist tropical plus (*MT+*) (*n* = 4). Each plot exhibits single-pollutant RR (NO_2_, O_3_, PM_2.5_, and SO_2_), plus an adjusted RR for the remaining air pollutants. Note *y*-axis scale differences between plots. A lack of PM_2.5_ and SO_2_ data resulted in insufficient data to run the model accurately in select cases for the hot weather types. *Asterisk* and *circumflex accent* represent a significantly increased or decreased adjusted effect estimate, respectively

Five graphs of the pooled single and adjusted pollutant models for all cities and weather types are presented in Fig. 1, representing RR due to air pollution exposure within 95 % CIs. The high proportions of significant risk estimates suggest a substantial health burden due to both single pollutant exposure (also in Table 4) and all two-way combinations of pollutants, in all five weather types. In all weather types, the single-pollutant effects of NO_2_, PM_2.5_, and SO_2_ on mortality decreased subsequent to adjustment for O_3_. This adjustment results in lower RR estimates in all cases, with 67 % being significantly less, and 40 % causing the significant pollution effect on mortality to disappear (Fig. 1). Additionally, adjusting for O_3_ caused the variability of the estimate to decrease in all but one of the cases, indicating better accuracy. Alternatively, results generally exhibit an increased variability in RR when the risk estimate increased after adjustment. Significant adjustments highlight the fact that the mortality effects of individual pollutants are not independent.

**Table 4:** Relative risk of mortality (RR) and 95 % CI associated with single pollutant models, calculated at pooled population weighted means (PWM), with standard error, for all cities combined

Weather type	Pollutant	PWM	SE	RR	95 % CI
DM	NO_2_	13.25	0.000	1.041^†^	(1.032–1.051)
	O_3_	12.58	0.000	1.032^†^	(1.023–1.041)
	PM_2.5_	12.99	0.001	1.050^†^	(1.035–1.065)
	SO_2_	12.05	0.001	1.059^†^	(1.042–1.076)
DT	NO_2_	12.01	0.001	1.067^†^	(1.035–1.100)
	O_3_	12.14	0.001	1.064^†^	(1.033–1.096)
	PM_2.5_	13.00	0.004	1.191^†^	(1.076–1.319)
	SO_2_	12.42	0.008	1.272^†^	(1.057–1.531)
MM	NO_2_	12.59	0.000	1.036^†^	(1.024–1.049)
	O_3_	11.99	0.000	1.036^†^	(1.026–1.045)
	PM_2.5_	12.80	0.001	1.050^†^	(1.034–1.065)
	SO2	13.11	0.001	1.041^†^	(1.027–1.055)
MT	NO_2_	14.22	0.001	1.038^†^	(1.019–1.057)
	O_3_	12.89	0.001	1.038^†^	(1.024–1.053)
	PM_2.5_	14.46	0.001	1.051^†^	(1.008–1.097)
	SO_2_	13.33	0.001	1.077^†^	(1.041–1.114)
MT+	NO_2_	13.44	0.002	1.117^†^	(1.053–1.186)
	O_3_	12.52	0.002	1.065^†^	(1.024–1.108)
	PM_2.5_	13.04	0.004	1.117^†^	(1.018–1.225)
	SO_2_	12.75	0.005	1.176^†^	(1.030–1.342)

^†^ *p* < 0.05, indicates statistical significance of the estimate

The risk estimates, however, are modified by weather type. For example, in the dry moderate (DM) weather type, the only case of dependence is found when adjusting the PM_2.5_ estimate for NO_2_; the RR estimate is 30 % lower (i.e., 1.020 (95 % CI 1.003–1.037)), which is a significant decrease. The remaining single pollutant RR estimates remain significant after adjustment for the remaining three air pollutants; hence, exposure to each pollutant alone has individual effects on mortality due to no significant change occurring after adjustment for another pollutant.

For the second moderate weather type—moist moderate (MM)—all single-pollutant exposure effects are statistically significant, each with magnitudes similar to those in the DM weather type. The significant effect of O_3_ individually, i.e., 1.036 (95 % CI 1.026–1.045), is not independent, as it decreases to an insignificant value after adjusting for PM_2.5_ (1.015 (95 % CI 0.990–1.032)). As these two pollutants demonstrate a significant and moderately positive correlation (*r* = 0.44) during MM weather (Table 2), we are evaluating a similar signal in the model. This also signifies that the single-model ozone RR value is an overestimate. A significant increase in the RR is found when PM_2.5_ is adjusted for SO_2_, where the estimate more than doubled (1.050 (95 % CI 1.034–1.065) versus 1.134 (95 % CI 1.059–1.215), respectively), and thus, the health effect of PM_2.5_ is dependent on SO_2_ concentrations. The NO_2_ mortality risk is found to be independent of the remaining pollutant levels, as all estimates remain significant after adjustments.

Within the MT weather type, all individual effects of air pollutant exposure on mortality are statistically significant, with RR magnitudes similar to those of MM and DM. The NO_2_ and O_3_ individual effects disappear after adjustment for SO_2_ and PM_2.5_, with a significant reduction of 3.5 % in RR present after adjusting NO_2_ for O_3_. The significant mortality effect due to PM_2.5_, i.e., 1.051 (95 % CI 1.008–1.097), becomes less variable and insignificant after adjusting for O_3_ (1.018 (95 % CI 0.996–1.041)). As these two pollutants display a significant and moderately positive correlation (*r* = 0.66), we are once again evaluating a similar signal, and the PM_2.5_ independent estimate may be significantly overestimating the health effect attributed to PM_2.5_.

In the DT and MT+ weather types, exposure to all air pollutants is found to have a stronger effect on mortality than in DM, MM, and MT weather, yet due to the lower number of cities experiencing DT and MT+ days (*n* = 5 and *n* = 4, respectively) and a low DT frequency, we see increased variability in the model output, as displayed by wide CIs. The single-pollutant exposure effects of all pollutants in the extreme DT weather type are statistically significant. The O_3_ and PM_2.5_ effects on mortality are independent in DT weather, as they remain statistically significant after adjustment for the remaining pollutants. This holds true even with significant declines in RR when PM_2.5_ is adjusted for by NO_2_ and O_3_. However, the NO_2_ effect is not independent, as it is significantly increased by adjustment for SO_2_.

For MT+, all single models are significant predictors of mortality. The only pollutant to display an independent effect on mortality is SO_2_, as the RR is not significantly altered by pollutant interactions. The NO_2_ effect significantly decreases and disappears when adjusted for both O_3_ and SO_2_. Sulfur dioxide displays the largest association with mortality, yet high variability (RR = 1.176 (95 % CI 1.030–1.342)) with O_3_ adjustment also resulting in a significant effect-size reduction, decreased variability, and an overall final insignificant effect. The absence of MT+ days in Canada and fewer years of PM_2.5_ data results in the model being unable to properly complete adjustments for PM_2.5_.

**Fig. 2:**
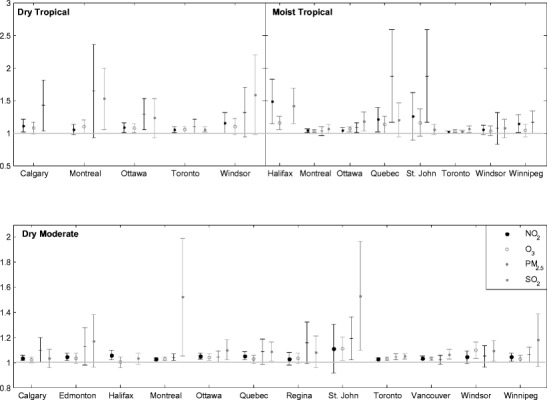
City-specific relative risks of mortality (RR) with 95 % confidence intervals (CI) within dry tropical (DT), moist tropical (MT), and dry moderate (DM) weather types. Each plot exhibits single-pollutant RR for NO_2_, O_3_, PM_2.5,_ and SO_2_. Note *y*-axis scale differences between plots

The summertime city-specific effects demonstrate differing associations between pollution and mortality risk. This variability among the 12 cities is illustrated using the three weather types of DM, DT, and MT in Fig. 2. The estimate variability is greater in the MT and DT weather types and less in DM weather. In Toronto, the risk estimates are significant for all four pollutants in all weather types, with very low variability. Similarly, in Ottawa, all RR estimates are significant for the three weather types due to all pollutants, with low variability (excluding the SO_2_ effect within DT air). Conversely, results for DM air in Regina and MT air in Windsor and Winnipeg are consistently insignificant. The greatest variation among cities occurs with PM_2.5_ and SO_2_, with very high variability in DT weather in Montreal and Windsor and in MT weather in Quebec and St. Johns. Lack of pollution data, mortality values, or select weather types contributes to such wide confidence intervals. On average, for all cities, the DM, MM, and MT weather types result in moderate increases in RR for exposure to all pollutants combined (RR = 1.045, 1.041, 1.051, respectively). However, within the DT or MT+ weather types, the all-pollutant average RR estimates are 14.9 and 11.9 % above expected risk, respectively, with SO_2_ found to be the most harmful pollutant in the hot summertime weather types.

## Discussion

Our findings complement the substantial evidence for negative health outcomes attributable to exposure to air pollution. The effect of atmospheric conditions on human health is expected to change with increasing magnitudes and frequencies of extreme weather as our climate changes (Gosling et al. 2007; O’Neill and Ebi 2009). Thus, increasing frequencies and intensity of specific weather types that negatively affect human health (i.e., “oppressive” weather types) will also occur (e.g., Greene et al. 2011; Knight et al. 2008; Vanos and Cakmak 2013; Vanos et al. 2014a; Vanos et al. 2014b); within which, the ambient effects to human health are vital to understand. There is a large variation in weather-type frequencies among large Canadian cities, which leads to a spatial variation in air pollution levels and health response. Further factors may include, but are not limited to, sources of pollution (industrial, vehicle, nearby areas), climate, topography, demography, time spent indoors, and socioeconomic factors. Therefore, one large-scale model with general criteria will not accurately estimate health outcomes in all cities, and the use of city-specific models is strongly suggested.

Within the current study, it is first established that extreme pollution episodes are significantly more likely in the hot weather types, being the strongest during dry tropical weather. Given these higher concentrations, we next sought out to determine if we can attribute these levels to a similarly greater risk in all-cause mortality within these weather types, making use of a DLNM approach. The modeled single-pollutant risk estimates are consistent with extreme episode findings in the hot, oppressive weather types. However, strong and statistically significant estimates are also present in the DM weather type. Such significant weather type modifications demonstrate the importance of categorizing the analysis into the weather types accounting for six meteorological variables, rather than one. For example, when comparing the moderate (DM and MM) weather types to the hot weather types (DT and MT+), we see that the average single-model RR due to PM_2.5_ triples (1.050 versus 1.154), and risk due to O_3_ almost doubles from 1.034 to 1.064. However, although NO_2_ and SO_2_ display little correlation with air temperature, the same changes also occur, whereby the RR estimates due to the NO_2_ and SO_2_ doubled and tripled, respectively, when comparing moderate and hot weather types.

Significant alterations (most commonly a decrease) of single pollutant risk estimates are found when a two-way pollutant adjustment is employed. The strong correlations between select pollutants and temperature result in difficulty isolating the causal signals (to the model) of one pollutant from signals of another or from weather elements. For example, Anderson and Bell (2009) found hot-weather effect estimates to be reduced when ozone was controlled for, demonstrating that ozone partly controls the mortality signal from the model. In the current study, significant modifications were not segregated to merely correlated pollutants or air temperature, as adjustment for ozone (displaying moderate correlation with PM_2.5_ only) had the highest frequency of significant estimate reductions.

This was a very evident trend in the results, displaying an overestimation of mortality risk by the pollutants NO_2_, SO_2_, and PM_2.5_ prior to adjustment for O_3_. This resulted in the significant single effect of the former pollutants disappearing. This demonstrates that ozone significantly controls a portion of the mortality signal from the model, agreeing with the temperature-effect finding stated above from Anderson and Bell (2009). The lone exception to this pattern occurred in the MM weather type for PM_2.5_ adjustment for O_3_, where a similar signal in the model may be coming from each of these pollutants due to their significant correlation in MM air; hence, ozone may be acting as a proxy for PM_2.5_. This also helps to explain the only instance of a significant reduction of the RR due to O_3_ when adjusted for PM_2.5_, within the MM weather type as well.

The improved accuracy in the O_3_-adjusted estimates is due to a strong independent signal from ozone. Adjusting effects for ozone concentrations may be the optimal method in this case to produce more accurate risk estimates and reduce potential overestimation of the remaining pollutants. The correlation of O_3_ with the remaining individual pollutants is very weak to zero (excluding PM_2.5_ adjustment in DT and MT weather, where *r* = 0.60 and 0.50, respectively). This was also found by Burnett et al. (2001) for O_3_ with SO_2_ and NO_2_ in Canadian cities. Previous studies have also demonstrated a relationship between ozone or respirable particles (PM) and mortality after adjusting for temperature (Hoek et al. 2000; Katsouyanni et al. 2001). Interactions between PM, O_3_, and high temperatures increase the relative mortality due to air pollution, more so under DT conditions. This attenuation of the RR was also demonstrated when adjusting particulate matter model contributions with gaseous pollutants (Burnett et al. 1995). Therefore, in the hot weather types, PM_2.5_ is not independent. Used alone, it is a less accurate indicator of RR and should be adjusted for O_3_ or other gaseous pollutants for more accurate mortality risk estimates.

The correlations and effect modification of NO_2_ by SO_2_ in hot dry air are corroborated by Burnett et al. (2004) in a study of 12 Canadian cities, as is ozone’s insensitivity to adjustment for PM_2.5_ (Burnett et al. 2001). These results agree with the insensitivity found in the moderate weather types in the current study, as well as SO_2_ estimates encompassing the full signal from PM_2.5_ in the DM weather type. Further, Brook et al. (2007) also found NO_2_ and PM_2.5_ to be significantly correlated in Canada, with NO_2_ being a better indicator of particles emitted from vehicles than PM_2.5_, as well as many other toxic indicator pollutants.

Assessing the results of extreme air pollution episode analysis together with the RR estimates (Table 3), we see that a high likelihood of extreme pollution does not always coincide with a high relative risk of mortality, particularly in the moderate weather types. This suggests that intervention strategies to lower the average levels of air pollutants on all days may be a more appropriate focus, rather than focusing on lowering peak levels (Schwartz 2000a; b). Health effects of weather due to hot weather types—DT, MT, and MT+—have frequently been associated with higher human mortality and morbidity due to heat exposure (Sheridan and Kalkstein 2004; Sheridan et al. 2009); however, the combined effects of heat and air pollution exposure are synergistic. In the DT, MT, and MT+ weather types, individuals experience extremely high temperatures (and humidity in the latter two) that may have health implications with respect to thermoregulation of blood flow, thereby resulting in heat-related mortality or morbidity (McGeehin and Mirabelli 2001). Therefore, there is a negative effect modification from both heat exposure and air pollution. Hence, it becomes more difficult to tease apart the independent effects when air temperature is correlated to air pollution, such as ozone, which demonstrates exposure risk estimates that are much greater in the two extreme weather types (DT and MT+).

Ozone is one of the most harmful air pollutants and has been shown to aggravate respiratory health problems (Hajat and Haines 2002). Based on future changes in atmospheric emissions, plus rising urban air temperatures consistent with climate change, Bell et al. (2007) found large average increases in levels of this pollutant across the USA, which corresponded to increased daily mortality. Further, Séguin and Berry (2008) state that ozone levels will increase the most in Windsor, Montreal, Toronto, Vancouver, Calgary, Edmonton, and Winnipeg. If we can accurately forecast approaching weather types associated with the probabilities of extreme heat *and* air pollution several days in advance (i.e., through the use of synoptic-based warning systems as in Sheridan and Kalkstein (2004)), then warnings can be issued to the general public, emergency personnel, hospitals, care givers, and others to save lives (Ebi et al. 2004). In this way, the detrimental effects of not only heat but also air pollution can be better managed. Such a synoptic-based warning system (derived from SSC) is currently used in Toronto and has shown promise as a pilot system for Canada to issue extreme heat alerts (Environment Canada 2012). Improved knowledge of the combined effects of temperature and air pollution on human mortality is vital for the medical community, policy makers, and community leaders to implement proper intervention strategies (Anderson and Bell 2009).

### Limitations

The assumption of equal exposure of all individuals to the same air pollution concentrations collected from a central site monitor is a limitation. This issue can only be resolved through the use of personal exposure monitors, which is a large endeavor for large populations. Questions regarding the amount of personal exposure to air pollutants, such as PM_2.5_, and the associations reported in daily time series analyses have been addressed in the literature (Schwartz 2000a; b). We also did not estimate the mortality displacement (or “harvesting effect”), where life is reduced by only a few days or weeks because of these environmental insults (Hajat et al. 2005); accordingly, our estimates of the true effect of air pollution on mortality may be slightly high. In addition, many subjective decisions must be made when estimating mortality due to air pollution, such as which confounding variables to adjust for, the shape of the exposure-response curve, lag structure, and temperature metric. This leads to difficultly in making study comparisons. A limitation of using longer lag structures is the introduction of more measurement error due to the increased time from exposure to event (Anderson and Bell 2009). Last, we defined extreme pollution events based on exposure to one pollutant only; however, in the future, a similar method can be employed that assesses the *mixture* of gas- and particulate-phase pollutants, which together can modify the effect on human health outcomes such as mortality.

## Conclusions

Our findings suggest that the health effects due to air pollution exposure differ under specific synoptic weather patterns and that pollutant interactions can significantly affect the relationships. As both individual and adjusted-pollutant models display statistically significant increases in RR due to all air pollutant exposures and under all weather types, attention must be given to all situations. Individual risk estimates are found to be significantly greater in the DT weather type for SO_2_ and PM_2.5_ and in the MT+ weather type for SO_2_, NO_2_, and PM_2.5_. However, when adjusted for interactions, the pollutants of NO_2_, SO_2_, and PM_2.5_ are found to not act independently to affect human health, as adjustment for O_3_ caused the overall RR estimates to decrease in magnitude (hence lessening the likelihood of over prediction) yet improve the accuracy, with insignificant estimates resulting at times. This demonstrates that ozone significantly controls a portion of the mortality signal from the DLNM model. Further, extreme air pollution episodes are more likely within the oppressive and hot synoptic categories of MT, MT+, and DT. As synoptic categories can be forecast several days in advance, then city-specific air pollution estimates can then be made for extreme pollution episodes and for issuing heat/health warnings.

Our findings highlight the synergistic effects of multiple air pollutants and weather impacting human health. Results complement the already substantial evidence of associations between air pollution and human health and should alert environmental policy makers to devote increased attention to weather-air pollution synergies and their effects on human health. Further research in Canada involving the specific cause of mortality and age effects using DLNM modeling can provide more targeted results on which to base stronger adaptation and implementation strategies. This is vital as all four pollutants were found to be associated with a significant increased risk of mortality in the general population for all five weather types studied, with even greater anomalies in the oppressive weather types. This study builds upon and complements existing SSC health studies by Health Canada and others across North America and will further aid in developing improved and advanced public health warnings and advisories to air pollution, during both extremely hot as well as dry moderate weather types.

## Abbreviations

AIC: Akaike’s information criteria
DLNM: Distributed lag nonlinear model
DM: Dry moderate
DP: Dry polar
DT: Dry tropical
GLM: Generalized linear model
MM: Moist moderate
MP: Moist polar
MT: Moist tropical
NO_2_: Nitrogen dioxide
O_3_: Ozone
PM_2.5_: Particulate matter <2.5 micrograms
SO_2_: Sulfur dioxide
RR: Relative risk of mortality
